#  The Effect of PEG Molecular Weights on Dissolution Behavior of Simvastatin in Solid Dispersions 

**Published:** 2013

**Authors:** Noushin Bolourchian, Mohammad Mehdi Mahboobian, Simin Dadashzadeh

**Affiliations:** a *Department of Pharmaceutics, School of Pharmacy, Shahid Beheshti University of Medical Sciences, Tehran, Iran.*; b *Pharmaceutical Sciences Research Center, Shahid Beheshti University of Medical Sciences, Tehran, Iran. *

**Keywords:** PEG molecular weight, Simvastatin, Solid dispersion, Dissolution, Solubility

## Abstract

The purpose of the present study was to investigate the effect of polyethylene glycol (PEG) molecular weights (6000, 12000 and 20000) as solid dispersion (SD) carriers on the dissolution behavior of simvastatin. SDs with various drug : carrier ratios were prepared by solvent method and evaluated for dissolution rate. Differential scanning calorimetry (DSC), X-ray diffraction (XRD), infrared spectroscopy and solubility studies were also performed on the optimum SD formulation. Samples prepared with all three types of PEG showed improved drug dissolution compared to intact drug and corresponding physical mixtures. Meanwhile, the best result was obtained by PEG 12000 with drug : carrier ratio of 1:7 which showed a 3-fold increase in dissolution rate compared to the intact drug. Based on DSC and XRD, no crystalline change occurred during the sample preparation. Solubility studies revealed that increasing the PEG molecular weight resulted in higher phase solubility of drug. In addition, saturated solubility of the optimum SD was significantly higher than that of intact drug and the related physical mixture (24.83, 8.74 and 8.88 μg/mL, respectively) that could be due to the decreased particle size and aggregation. The results confirmed the influence of PEG molecular weight on drug dissolution rate from solid dispersion systems.

## Introduction

Simvastatin (SIM) is a 3-hydroxy-3- methylglutaryl-coenzyme, a reductase inhibitor which is mostly used in the treatment of hypercholesterolemia (1). As a BCS (Biopharmaceutics Classification System) class II drug, it is water-insoluble (2) and shows dissolution rate-limited absorption with low oral bioavailability (3, 4). Therefore, increasing the dissolution rate of simvastatin could lead to improve its bioavailability and better therapeutic efficacy. 

Among the various approaches of poorly water-soluble drug dissolution enhancement, solid dispersion (SD) of drug in a water-soluble carrier is one of the effective methods (5-11). SD is defined as a dispersion of one or more active ingredients in an inert carrier, usually highly water-soluble compound, which could be prepared by different methods including melting, solvent and melting-solvent techniques (12, 13). Different mechanisms are considered for drug solubility and dissolution rate enhancement using SDs. Particle size reduction of drug to submicron size and consequent increase in surface area seems to be one of the major factors (14). An increase in the saturation solubility through the means of reducing the particle size to less than 1 μm has also been reported (15). Decreasing in particle aggregation and agglomeration as well as improved wettability of drug particles by direct contact of drug with the hydrophilic carrier are other influencing factors (16). Moreover, changing the drug from crystalline to amorphous state with higher energy can increase the drug solubility (17).

Some investigations have been carried out regarding the improvement of simvastatin dissolution through solid dispersion preparation. Rao *et al. *used superdisintegrants as hydrophilic carriers to prepare SDs by co-evaporation method and achieved better dissolution rate of simvastatin due to decreased crystallinity of drug (4). In two separate studies it was shown that preparation of SIM solid dispersions with PEG or PVP could be a promising strategy to improve dissolution rate and therefore the bioavailability of the drug (18, 19). However, SIM solid dispersion with PEG was more advantageous over the dispersions prepared with PVP since they did not show drug degradation during the preparation (19).

PEG as a hydrophilic carrier is used commonly in the preparation of SDs formulation and is available in a range of molecular weights (20-26). Each molecular weight of the polymer has definite characteristics that may lead to various characteristics of SD formulations.

Some previous studies have investigated the effect of PEG molecular weights on the dissolution rate of drugs from SDs formulations but the results are not completely consistent with each other. Narang and Srivastava did not find any definite relationship between the PEG molecular weights and the release rate of clofazimine from SDs (27), while study of the dissolution of itraconazole from SDs containing PEG-HPMC (hydroxypropyl methylcellulose) showed that in lower percentages of PEG, shorter chain length had higher dissolution rate of drug compared to long chain length PEG type. This difference was not observed in formulations with higher PEG-HPMC ratios (28). Ozkan *et al. *showed that in etodolac : PEG molar ratio of 1:5 SD, PEG 6000 increased the drug dissolution to a great extent, compared to higher and lower molecular weights (29). On the other hand PEG 8000 was more effective in dissolution rate enhancement of etoposide rather than PEG 6000 (30).

To our knowledge, no study has been carried out regarding the effect of PEG molecular weights on simvastatin SD. In the light of this controversy, the aim of the present study was to investigate the effect of low and high molecular weight PEGs as solid dispersion carriers on the physical properties and dissolution behavior of simvastatin, a poorly soluble drug.

## Experimental

Simvastatin was obtained as a gift form Shahr darou Laboratories Co. Ltd (Shahr Darou, Iran). PEG 6000, 12000 and 20000 were purchased from Fluka (Fluka, Germany). All other chemicals and solvents used were of pharmaceutical grades.

*Preparation of solid dispersions*

In order to prepare SDs by solvent method, the sufficient amount of simvastatin and carriers were dissolved in ethanol with constant stirring for 30 min. The mixture was kept in the oven for 24 h at 40°C and then at room temperature for 48 to 72 h until a dry cake was obtained. Dry batches were sieved and particles that remained between 80 and 120 mesh were selected for the next experiments. SDs were prepared using SIM and different molecular weights of PEG (6000, 12000 and 20000) in various ratios of 1:1, 1:3, 1:5, 1:7 and 1:9.

*Preparation of physical mixtures*

Physical mixtures (PMs) were prepared by manually mixing of SIM and different PEGs which were previously sieved through 80 and 120 meshes in a mortar for 5 min, until a homogenous mixture was obtained.

*In vitro dissolution studies*

Dissolution rate of samples (SDs, PMs and untreated SIM) was studied in 500 mL phosphate buffer solution (pH = 6.8) containing 0.01% SLS at 37°C at a rotation speed of 75 rpm for 1 h using USP type I (basket) dissolution test apparatus (Erweka DT6R, Germany). At various time intervals, 2 mL of the medium was taken and replaced with the same volume of fresh buffer. Samples were analyzed spectrophotometrically for SIM content at 238 nm (Shimadzu UV 1201, Japan).

Since the higher solubility of drug in the dissolution medium can not make a good distinction between the different formulations, a pseudo-sink condition was selected to perform dissolution tests in this study (16).

Moreover, for more precise evaluation, the dissolution test was carried out for the optimum sample and related physical mixture in the medium containing 0.1% SLS in order to provide sink condition.

*Dissolution data analysis*

Dissolution efficiency (DE30) was calculated based on the area under the dissolution curve from 0 to 30 min, measured by trapezoidal rule, expressed as a percentage of the area at maximum dissolution (31).

*Powder X-ray diffraction (XRD)*

To evaluate the crystalline properties of prepared samples, XRD pattern was measured by a Philips Xpert diffractometer (The Netherlands) over the range of 2-70^o^ 2θ using Cukα radiation with the scan rate of 1°/min.

*Differential scanning calorimetry (DSC)*

DSC thermograms were recorded using a Shimadzu DSC-60 calorimeter (Shimadzu, Japan). About 5 mg of each sample was heated over the temperature range of 20-220°C with the rate of 10°C/min. An empty pan was also used as a reference.

*Infrared spectroscopy (IR)*

About 2-3 mg of selected samples was triturated with potassium bromide and compressed into a disc (12 mm) at 10 ton pressure. IR adsorption spectra were recorded using a spectrometer (Perkin-elmer 843, UK) over a range of 200-4000 cm^-1^.

*Saturated solubility*

Samples (SIM, SDs and PMs) containing excess amounts of SIM were added to 10 mL distilled water and kept under magnetic stirring for 24 h at 25 ± 0.5°C. Then, they were centrifuged and analyzed for SIM content at 238 nm (Shimadzu mini UV 1240, Japan). This test was carried out in triplicate for each sample.

*Phase solubility*

Aqueous solutions (10 mL) containing increasing concentrations (2.5, 5, 7.5 and 10% W/V) of each polymer (PEG 6000, 12000 and 20000) were prepared and then excess amount of drug was added and kept under magnetic stirring for 24 h at 25 ± 0.5°C. Samples were centrifuged and assayed spectrophotometrically at 238 nm.

*Statistical analysis*

All data obtained from dissolution and solubility studies were compared using analysis of variance (ANOVA) with Tukey post test.

## Results and Discussion

*Dissolution studies at pseudo-sink conditions*

**Figure 1:**
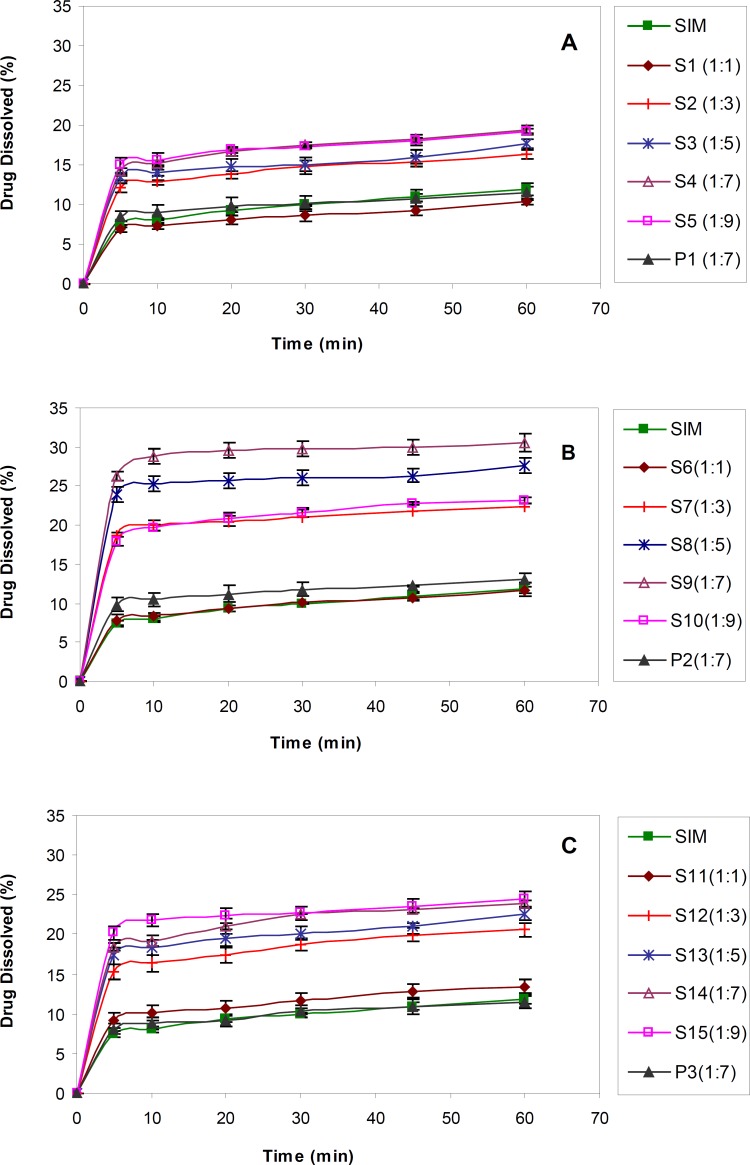
Dissolution profiles of untreated drug (SIM), PMs, SDs with different drug : polymer ratios, A) SIM : PEG 6000, B) SIM : PEG 12000 C) SIM : PEG 20000 (n = 3).

The dissolution profiles obtained for untreated SIM and SIM SDs prepared in drug : carrier ratios by using various molecular weights of PEG were shown in Figure 1.

 For all molecular weights of PEG, a lower drug : carrier ratio (1:1) did not show any influence on drug dissolution rate enhancement, due to the lower solubility of SIM. On the contrary, dissolution rate of drug from SDs in the ratios of 1:3 to 1:9 was obviously increased in comparison with that of untreated SIM. As shown in Table 1, there was a significant difference (p < 0.05) between DE30 obtained for S2-S5 (prepared by PEG 6000) compared to the untreated drug. 

**Table 1:** Composition of samples (SDs and PMs) and the dissolution efficiencies (DE_30_).

**Sample**	**Drug:polymer ratio**			**DE**30 **(%) (n = 3)**
	**PEG 6000**	**PEG 12000**	**PEG 20000**
**SIM** ^a^	1:0	-	-	8.10 ± 0.18
**S1** ^b^	1:1	-	-	7.10 ± 0.45
**S2**	1:3	-	-	12.28 ± 0.35
**S3**	1:5	-	-	13.18 ± 0.51
**S4**	1:7	-	-	14.64 ± 0.24
**S5**	1:9	-	-	14.92 ± 0.44
**P1** ^c^	1:7	-	-	8.64 ± 0.93
**S6**	-	1:1	-	8.14 ± 0.33
**S7**	-	1:3	-	18.37 ± 0.61
**S8**	-	1:5	-	23.23 ± 0.87
**S9**	-	1:7	-	26.37 ± 0.83
**S10**	-	1:9	-	18.37 ± 0.40
**P2**	-	1:7	-	9.90 ± 0.85
**S11**	-	-	1:1	9.58 ± 0.87
**S12**	-	-	1:3	15.54 ± 0.83
**S13**	-	-	1:5	17.36 ± 0.86
**S14**	-	-	1:7	18.64 ± 0.50
**S15**	-	-	1:9	20.07 ± 0.81
**P3**	-	-	1:7	8.34 ± 0.72

^a^SIM: Untreated drug; bS: Solid dispersion; cP: Physical mixture

The same results were achieved for SDs prepared using PEGs 12000 and 20000. The DE30 values obtained for drug : carrier ratios equal to 1:7 (S4, S9 and S14) were significantly higher than formulations prepared with lower amount of PEG, although they were similar to or even better than the results achieved for 1:9 ratios (S5, S10 and S15). In fact, increasing the PEG concentration in SD formulations only to a specific amount resulted in better drug dissolution and further polymer increasing, did not show any additional improvement. This might be attributed to the viscous layer formed around the solid particles due to higher PEG concentrations and therefore decreased the diffusion coefficient (based on Stokes-Einstein equation) and lower drug dissolution (32). As a result, SDs with drug : carrier ratios of 1:7 were considered as the best formulations compared to untreated SIM as well as other drug : carrier ratios.

In order to find out the effect of polymer itself on drug dissolution, PMs containing drug and polymer (1:7) were prepared and subjected to dissolution test (Figure 1). The results proved that SIM dissolution from PMs was almost similar to the intact drug (Table 1). In fact, the solid dispersion process had a great effect on drug dissolution enhancement probably due to the drug particle size decreasing through the process.

With regard to the effect of the type of polymer, based on the results, PEG 12000 had better influence on the drug dissolution compared to two other PEG molecular weights. The dissolution efficiency calculated for S9 (with PEG 12000) was significantly higher than S4 and S14 (with PEG 6000 and 20000, respectively) (p < 0.001). It seems that highly viscous solution appeared around the drug particles due to PEG 20000 could inhibit the dissolution enhancement to some extent, whereas, lower hydrophilicity of PEG 6000 compared to PEG 12000 might be the main cause of its slower drug solution. Therefore, the dissolution rate enhancement of poorly soluble drugs from SDs seems to be affected by various factors, including the hydrophilicity of soluble carrier and the viscosity of the medium around the particles. In order to obtain desirable results, a balance must be achieved between those two factors.

In general, the S9 sample, composed of PEG 12000 with the drug : carrier ratio of 1:7, showed a 3-fold rise in simvastatin dissolution rate and therefore was selected as the best sample among all other batches.

*Dissolution studies at sink conditions*

**Figure 2:**
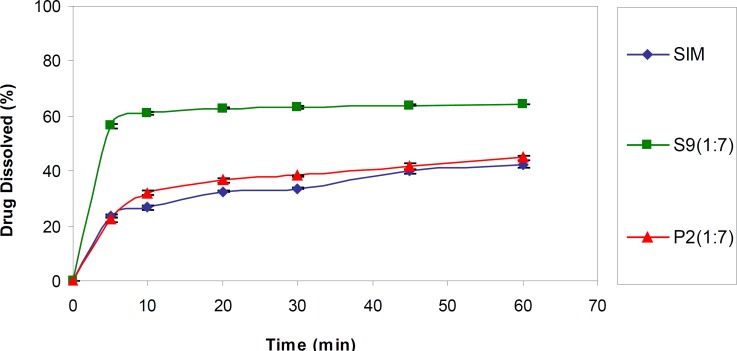
Dissolution profiles of untreated drug (SIM), selected SD (S9) and PM (P2) in the medium containing 0.1% SLS (n = 3).

For better evaluation, in the next step, S9 (the optimum formulation) was subjected to dissolution test in a sink condition. As shown in Figure 2, the drug dissolution rate from this SD sample was significantly higher (p < 0.001) than the intact drug as well as corresponding PM (P2) (DE30 = 56.09 ± 0.30, 27.07 ± 0.26 and 30.039 ± 0.43%, respectively). Although the application of higher SLS concentration in the dissolution medium resulted in elevated drug release rate compared to the pseudo-sink medium, as it is obvious, the observed difference between the samples was declined in the sink conditions compared to the pseudo-sink medium.

*Powder X-ray diffraction (XRD)*

**Figure 3:**
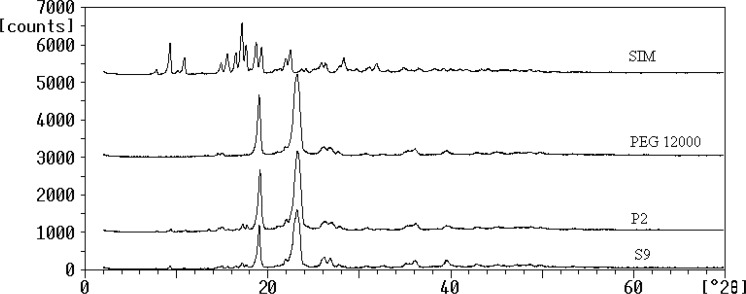
XRD patterns for the untreated drug (SIM), PEG 12000, selected SD (S9) and PM (P2).

The powder XRD patterns of SIM, PEG 12000, selected SD (S9) and the corresponding PM were depicted in Figure 3. Several distinct peaks were observed at diffraction angles (2θ) of 9.3, 10.9, 17.1, 18.75, 22.5 and 23.29 for SIM and also 19.9 and 23.24 for PEG 12000 which indicated their crystalline properties (18, 31). The XRD peaks of untreated SIM could be detected in S9 and P2 at the same 2θ values which confirmed that drug remains crystalline in solid dispersion and physical mixture and no crystallinity changes (amorphization) occurred during the preparation process. However, the intensity of the peaks of SIM in both samples was significantly less than that of untreated drug, which could be attributed to the lower percentage of drug incorporated in samples preparation compared to the carrier. Based on this result, the enhancement of SIM dissolution from S9 was not related to the crystalline changes and could be due to the other factors mainly particle size reduction.

*Differential scanning calorimetry (DSC)*

**Figure 4:**
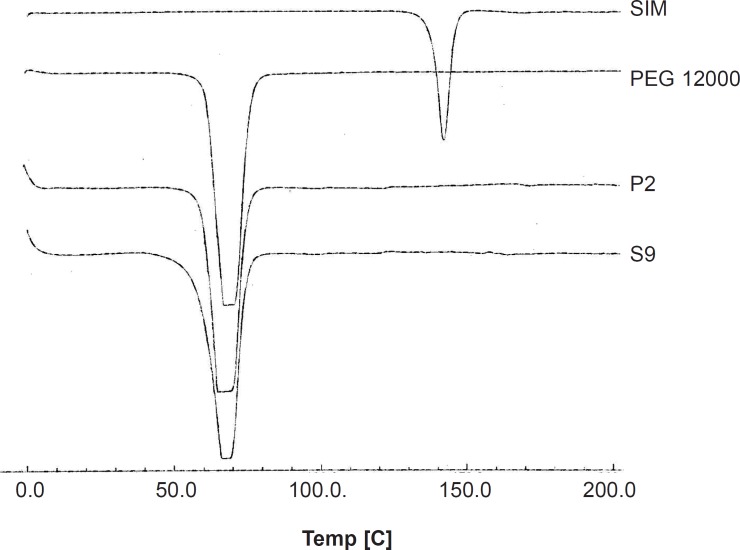
DSC thermograms for the untreated drug (SIM), PEG 12000, selected SD (S9) and PM (P2).

DSC thermograms of selected solid dispersion and related physical mixture as well as PEG 12000 and intact drug are illustrated in Figure 4. Endothermic peaks with the onset temperature of 137.95 and 60.66°C were exhibited for the intact SIM and PEG, respectively, corresponding to the melting point of each component. The drug melting point was completely disappeared in both thermograms obtained for SD and PM of simvastatin with drug : carrier ratio of 1 : 7. Only one peak could be observed in both curves which was related to PEG. In general, disappearance of drug melting endothermic peak could be attributed to the lack of crystalline structure of drug in the sample or the solubility of drug in melted carrier during the test. Since in the previous section, the XRD spectra proved that drug was in crystalline and not amorphous state in SD sample, so the second condition might have been occurred during the DSC analysis. The same thing could be happened for PM sample in the DSC pan.

*Infrared spectroscopy (IR)*

**Figure 5:**
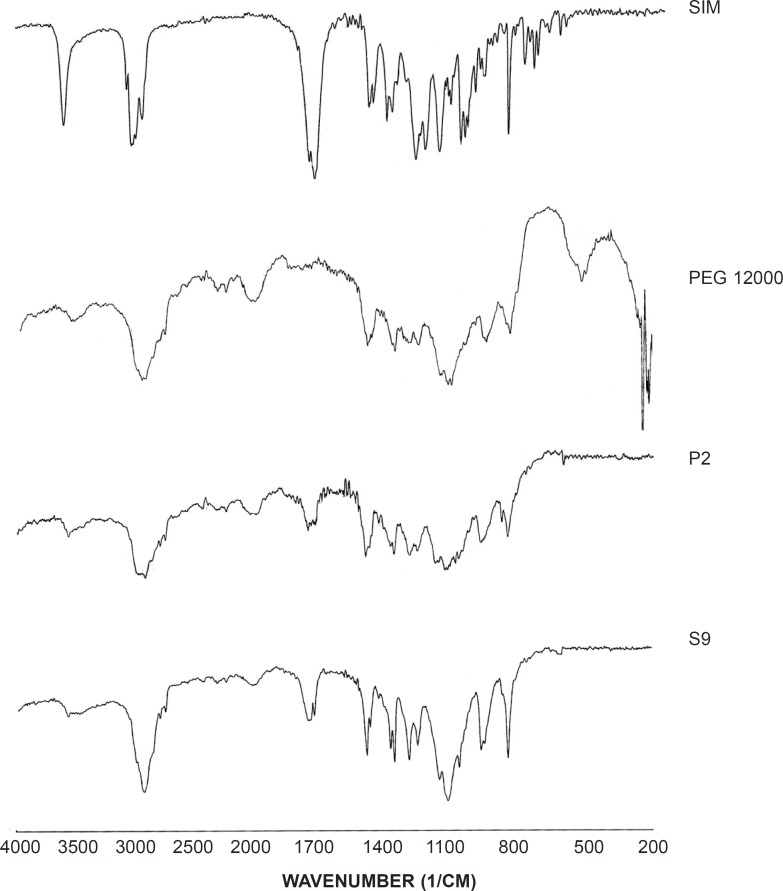
IR spectra for the untreated drug (SIM), PEG 12000, selected SD (S9) and PM (P2).

IR spectroscopy was performed in order to identify any possible interaction between the drug and the carrier in SD formulation. Figure 5 demonstrates the IR spectra of the intact drug, the polymer, SD and corresponding PM (1:7). The spectrum of pure SIM presented characteristic signals at 1277 cm^-1^ (C-O stretching vibration), 1401 and 1477 cm-1 (CH3 bending vibration), 1709 cm^-1^ (carbonyl stretching vibration), 2988 cm^-1^ (C-H stretching vibration) and 3570 cm^-1^ (O-H stretching vibration). In addition, the main peaks of PEG 12000 were appeared at 1117, 2908 and 3499 cm^-1^ which were related to the C-O stretching, C-H stretching and O-H stretching vibrations, respectively. Since the main signals of the drug and the carrier appeared in SD and PM samples, no interaction seems to be occurred in both sample preparations.

Comparing the SD and PM spectra and the untreated drug, a broad peak of O-H stretching vibration with a slight shift to lower wavenumbers could be observed in both S9 and P2 that indicates the possibility of intermolecular hydrogen bonding between the drug and the carrier (34).

On the other hand, CH3 bending vibration peak shifted to the lower wavenumber in both solid dispersion and physical mixture that might be attributed to the van der walls forces between SIM and PEG.

*Solubility studies*

Increasing the solubility of drug in the presence of hydrophilic carriers is considered as one of the mechanisms for drug dissolution rate enhancement (27). Phase solubility studies (Table 2) revealed that the presence of different concentrations of PEGs in the medium increased the water solubility of SIM significantly (p < 0.001). Meanwhile, PEG molecular weight had a great influence on this matter, the higher the molecular weight, the higher the phase solubility. This observation could be accounted by the higher hydrophilicity and water solubility of the high molecular weight PEGs. PEG 20000 is more hydrophilic than lower molecular weight PEGs and resulted in an increased SIM solubility in the medium. Therefore, solubilization of drug by the carrier could be considered as one of the mechanisms for SIM improved dissolution rate. 

**Table 2:** Phase solubility of SIM in the presence of different concentrations of PEGs in aqueous medium

**Polymer type**	**SIM solubility in the presence of increasing concentrations of PEGs (μg/mL) (n = 3)**				
	**0% PEG**	**2.5% PEG**	**5% PEG**	**7.5% PEG**	**10% PEG**	
**PEG 6000**	8.74 ± 0.13	16.63 ± 0.07	17.39 ± 0.05	17.91 ± 0.8	18.31 ± 0.08	
**PEG 12000**	8.74 ± 0.13	18.14 ± 0.07	21.25 ± 0.05	23.43 ± 0.07	27.11 ± 0.11	
**PEG 20000**	8.74 ± 0.13	28.25 ± 0.08	30.48 ± 0.08	39.36 ± 0.31	47.01 ± 0.20	

Based on Noyes-Whitney equation, the enhancement of saturated solubility of drug could improve its dissolution rate (35). Table 3 shows the saturated solubility data obtained for untreated SIM, SD and PM samples in water. The drug saturated solubility of selected solid dispersion was significantly higher than that of intact drug. Since the saturated solubility obtained for SD was higher than PM, it can be concluded that higher drug saturated solubility was not only related to the presence of polymeric carrier in the medium, but also is associated to the drug status in the solid dispersion system (reduced particle size and higher surface area).

**Table 3:** Saturated solubility of SIM, selected SD and corresponding PM

**Sample**	**SIM saturation solubility (μg/mL) (n = 3)**
**SIM**	8.74 ± 0.13
**S9**	24.83 ± 0.11
**P2**	8.88 ± 0.15

## Conclusion

Simvastatin dissolution rate was markedly improved in the form of solid dispersion using PEGs as hydrophilic carriers. The results confirmed that the carrier molecular weight has a noticeable influence on the drug dissolution rate from solid dispersion systems. It seems that polymer hydrophilicity as well as viscosity plays a significant role in the drug dissolution process. Reduced drug particle size, decreased particle aggregation and higher wettability of drug in the presence of hydrophilic carrier could be considered as the most probable mechanisms for enhanced dissolution rate of SIM from solid dispersion systems.
